# Prognosis and histology of sporadic synchronous and metachronous meningiomas and comparative analyses with singular lesions

**DOI:** 10.1007/s10143-023-01958-w

**Published:** 2023-02-13

**Authors:** Lisa Kopf, Nils Warneke, Oliver Grauer, Christian Thomas, Katharina Hess, Michael Schwake, Manoj Mannil, Burak Han Akkurt, Werner Paulus, Walter Stummer, Benjamin Brokinkel, Dorothee Cäcilia Spille

**Affiliations:** 1 Department of Neurosurgery, University Hospital Münster, Albert-Schweitzer-Campus 1, Building A1, 48149 Münster, Germany; 2 Department of Neurology, University of Münster, Münster, Germany; 3 Institute of Neuropathology, University Hospital Münster, Münster, Germany; 4 Department of Pathology, University Medical Centre Schleswig-Holstein, Campus Kiel, Kiel, Germany; 5 Clinic for Radiology, University Hospital Münster, Münster, Germany

**Keywords:** Meningiomas, Meningiomatosis, Multiple, Prognosis, WHO grade

## Abstract

Synchronous or metachronous growth of multiple tumors (≥ 2) is found in up to 20% of meningioma patients. However, biological as well as histological features and prognosis are largely unexplored. Clinical and histological characteristics were retrospectively investigated in 95 patients harboring 226 multiple meningiomas (MMs) and compared with 135 cases of singular meningiomas (SM) using uni- and multivariate analyses. In MM, tumors occurred synchronously and metachronously in 62% and 38%, respectively. WHO grade was intra-individually constant in all but two MMs, and histological subtype varied in 13% of grade 1 tumors. MM occurred more commonly in convexity/parasagittal locations, while SM were more frequent at the skull base (*p* < .001). In univariate analyses, gross total resection (*p* = .014) and high-grade histology in MM were associated with a prolonged time to progression (*p* < .001). Most clinical characteristics and rates of high-grade histology were similar in both groups (*p* ≥ .05, each). Multivariate analyses showed synchronous/metachronous meningioma growth (HR 4.50, 95% CI 2.26–8.96; *p* < .001) as an independent predictor for progression. Compared to SM, risk of progression was similar in cases with two (HR 1.56, 95% CI .76–3.19; *p* = .224), but exponentially raised in patients with 3–4 (HR 3.25, 1.22–1.62; *p* = .018) and ≥ 5 tumors (HR 13.80, 4.06–46.96; *p* < .001). Clinical and histological characteristics and risk factors for progression do not relevantly differ between SM and MM. Although largely constant, histology and WHO grade occasionally intra-individually vary in MM. A distinctly higher risk of disease progression in MM as compared to SM might reflect different underlying molecular alterations.

## Introduction

Meningiomas are the most common primary intracranial neoplasms and occur in multiple intracranial or spinal locations (≥ 2) in one individual in up to 20% [14, 19, 20, 31]. Etiology of MM growth, with either multiple distant lesions at the date of diagnosis (synchronous MM) or the development of spatially separated tumors during follow-up (metachronous MM), has been sparsely investigated. MMs are more commonly found following whole brain radiation therapy during childhood, e.g., due to leukemia, and in patients suffering from phacomatoses, especially neurofibromatosis (NF) 2. Pathophysiological mechanisms underlying tumor manifestations at different, distant sites remain largely obscure but eventually suggest both sporadic multiple tumor growth and leptomeningeal spread [19]. While shown to be associated with increased Ki67 proliferation indices [17], multifocal tumor growth is observed among meningiomas of all WHO grades [1]. However, comparative histopathological analyses of tumors arising in one individual are sparse [4]. Genetic and molecular alterations underlying MM growth are largely unexplored. Previous analyses in small series or case reports of MM revealed single mutations or chromosomal aberrations of *NF2* or *SMARCB1* as well as their monoclonal origin [10, 27, 30, 33]. In contrast, a recent study showed different intra-individual driver mutations in MM [10, 15, 23].

Beyond pathophysiology and pathogenesis, treatment of MM remains a key challenge during neuro-oncological care. Few previous studies showed increased recurrence rates in MM as compared to singular lesions, suggesting different biological behavior and worse overall prognosis [13, 20, 35]. In fact, disseminated tumor growth at different intracranial sites and multiple recurrences limit local treatment options such as microsurgical resection or radiosurgery. In addition, effective chemotherapeutical options for meningioma patients are lacking [5]. Hence, further characterization of the clinical course of patients with MM and identification of risk factors for progression are urgently needed.

In this series, we therefore analyzed histological and clinical characteristics of patients suffering from sporadic neuropathologically confirmed synchronous or metachronous meningiomas and additionally provide comparative analyses with individuals harboring singular intracranial lesions.

## Materials and methods

### Data collection

Imaging, medical, and operative reports from all patients who underwent surgery for neuropathologically confirmed meningioma in our department (Department of Neurosurgery, University Hospital Münster, Germany), between 1991 and 2018, were reviewed. MM was classified in case of ≥ 2 histopathologically confirmed meningiomas or meningioma-suspicious intracranial lesions at the date of index surgery (synchronous) or during follow-up (metachronous MM). Cases with multiple tumors arising from the resection cavity/dura attachment after surgery for a single lesion were not included. Radiological diagnosis of meningioma not subjected to surgery was established in patients with the history of at least one neuropathologically confirmed meningioma and distant, synchronously or metachronously growing contrast-enhancing, extra-axial lesions. In surgically treated cases, meningioma diagnosis and grading were performed according to the 2016 classification of brain tumors in all cases [18]. The following data was registered as described previously [2, 7]: patients’ sex and age at the time of index surgery, Karnofsky Performance Score (KPS, [11]) prior to index surgery and at the date of last follow-up, indication for surgery (primary or recurrent meningioma), tumor location (classified as “skull base,” “convexity” and “parasagittal/falcine,” “spinal” and “intraventricular”), administration of preoperative or adjuvant irradiation, and the extent of resection according to the Simpson classification system [25], dichotomously registered as gross and subtotal resection (GTR, Simpson I–III vs STR, Simpson ≥ IV, [2]) for each single lesion. For comparative analyses, a cohort of patients with singular meningiomas with a follow-up of at least 3 years and full availability of the included histological and clinical data was retrieved from the Muenster Meningioma Database [2, 7, 26] using computed randomized sampling (IBM SPSS Statistics, Version 28, IBM, Ehningen, Germany). No further restrictions were applied when defining the cohort.

Initial routine postoperative MRI was scheduled 3 months after surgery and, in case of an event-free course, repeated in 12- and 6-month intervals in grade 1 and 2/3 lesions, respectively. After a progression-free interval of 5 years, follow-up imaging intervals were extended to 24 months in grade 1 and 12 months in grade 2/3 lesions [5]. Contrast-enhanced CT scans were performed in patients with contra-indications against MRI, and imaging was analyzed by a team of at least one neurosurgeon and one radiologist. Recurrence/progression was registered for each tumor and diagnosed in case of any increase in tumor size beyond MRI- or CT-depending measurement range, while development of distant tumors qualified registration as metachronous MM. Time to progression was correspondingly calculated from the date of initial diagnosis of each lesion to the date of its progression.

### Statistical analyses

Statistical calculations were performed using statistic software (IBM SPSS Statistics, Version 28, IBM, Ehningen, Germany). Data are characterized by standard statistics: Categorical variables are described by absolute and relative frequencies and compared by Fisher’s exact test, while continuous variables are described by median and range and compared by Mann–Whitney *U* test. Time to progression (TTP) was defined as the duration between index surgery of surgically treated meningiomas or initial diagnosis of non-surgically treated lesions and radiologically confirmed tumor recurrence/progression or, in case of an event-free follow-up, to the date of last follow-up. TTP was analyzed by the Kaplan–Meier method and compared by Log-rank tests.

Multivariate Cox regression analyses for TTP included patients’ age, sex (female = reference (ref)), tumor location (convexity = ref), histology (grade 1 = ref; vs high-grade (grade 2/3)), and the extent of resection (GTR = ref). The results are characterized by hazard ratios (HR), 95% confidence intervals (CIs), and Wald-test *p* values. All reported *p* values are two-sided and considered statistically significant when < 0.05. Data collection and scientific use were approved by the local ethics committee (Münster 2018–061-f-S) and in accordance with the principles of the Declaration of Helsinki (human rights).

## Results

**Fig. 1:**
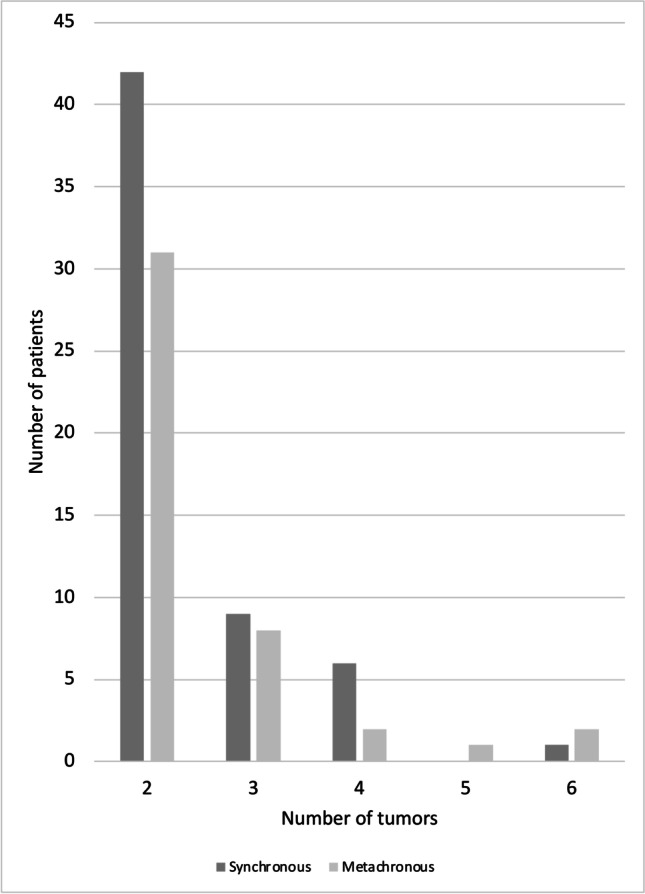
Numbers of lesions per patient in our cohort. The bar diagram displays an exponential decrease of the number of affected patients as the number of synchronous and metachronous tumors increases

Using the above-described approach, of 1404 patients in the Muenster Meningioma Database, 95 individuals (7%) with sporadic multiple meningiomas, harboring a total of 226 lesions (mean: 2.4 tumors per patient), were identified, while 5 patients suffering from NF1/2 and 6 individuals who received cranial irradiation during childhood were excluded. The number of patients decreased as the number of tumors per patient increased (Fig. 1). One hundred forty-one lesions were synchronously diagnosed at the date of presentation, while 85 tumors appeared metachronously during follow-up (62% vs 38%). Median duration between index surgery and the development of the following metachronous, distant lesion was 38 months. 132 tumors were subjected to microsurgery (59%), followed by adjuvant irradiation in 38 cases (29%). During the further clinical course, irradiation for tumor relapse or progression was performed in 15 lesions. Six patients received peptide receptor radionuclide therapy (PRRT), mostly with 177Lu-DOTATATE, while the remaining patients were subjected to observation imaging. In Table 1, the left column summarizes baseline clinical data of patients with multiple meningiomas.

**Table 1:**
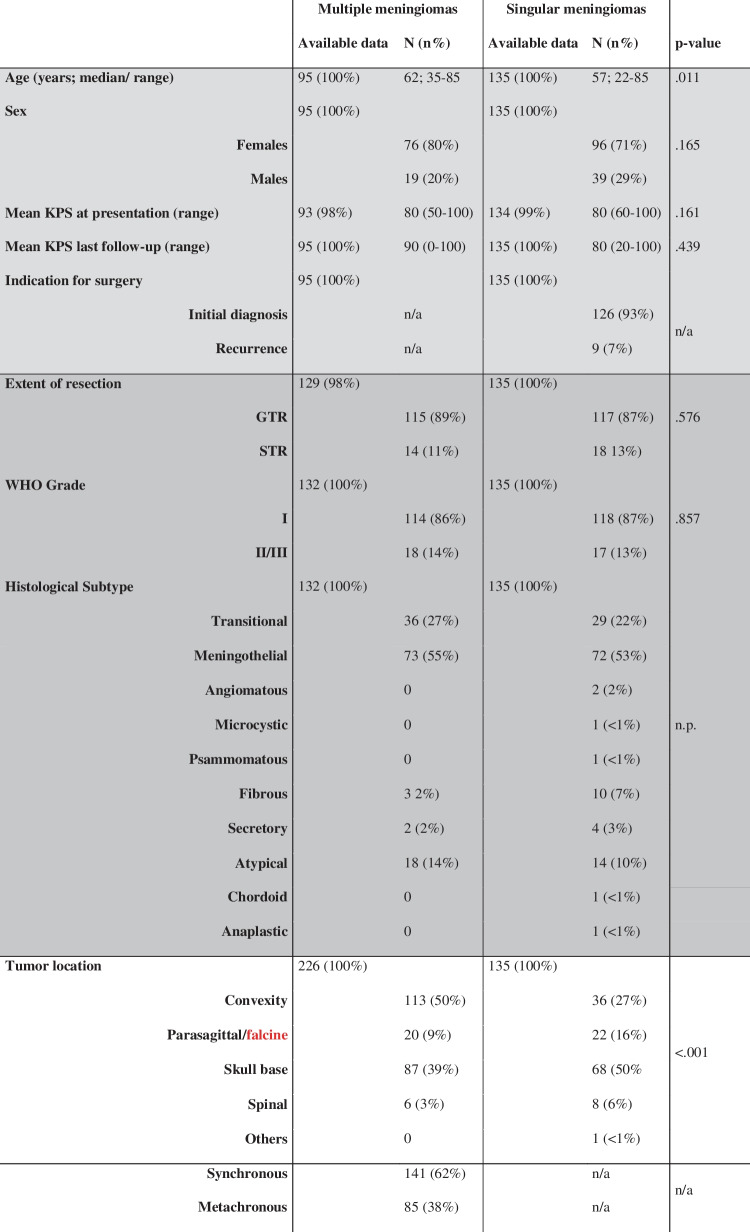
Baseline clinical and histological characteristics as well as comparative analyses of patients with multiple (MMs) and singular meningiomas (SMs). Data has been available in the vast majority of cases. Reference number of MM cases vary among the analyzed variables: Clinical data at the date of index surgery are patient-specific (*N* = 95, light gray), extent of resection and histological data is only reported for surgically treated tumors (*N* = 132, medium gray), and the tumor location is reported for all MM lesions (*N* = 226, white)

### Histology and clinical characteristics of patients with MM

**Fig. 2:**
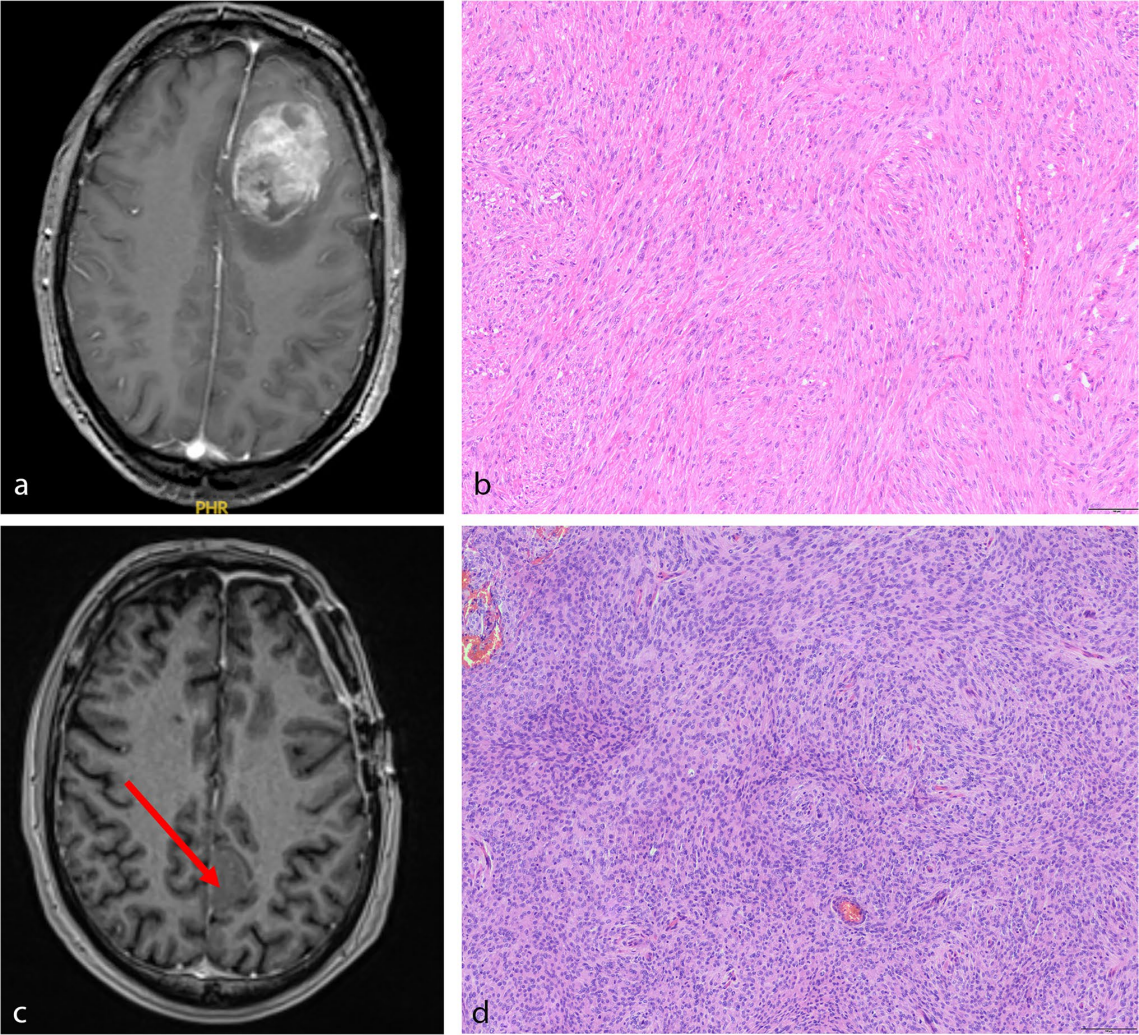
Illustrative sample of a patient developing metachronous spatially distinct meningiomas. After resection of a left frontal parasagittal meningioma (**a**, axial T1-weighted, contrast-enhanced imaging), microscopic analyses revealed transitional meningioma, WHO grade 1, **b**). Four years later, the patient developed a left parietal, parasagittal lesion (axial T1-weighted, contrast-enhanced imaging, **c**, arrow), diagnosed as atypical meningioma (**d**, hematoxylin and eosin staining, each)

High-grade histology was diagnosed in 18 of the operated 132 MM (14%) and was found in nine of 85 synchronous and nine of 47 metachronous lesions (11% vs 19%, *p* = 0.192). Similarly, high-grade histology was not related with tumor location (*p* = 0.099) or KPS at the time of initial or last presentation (*p* = 0.295). However, mean KPS at the date of the last follow-up was lower in patients with high-grade meningiomas than in individuals with benign lesions (80, SD ± 20 vs 90, SD ± 10 vs, *p* = 0.025). Noteworthy, neuropathological analyses revealed a constant WHO grade among all analyzed samples except two patients. Those included two male patients, one suffering from six tumors, subsuming one benign, three high-grade, and two non-operated meningiomas (see illustrative Fig. 2), and another male suffering from three meningiomas, subsuming one benign, one high-grade, and one not operated lesion. In 56 samples of 25 patients with multiple grade 1 meningiomas who underwent surgery, neuropathological analyses further showed intra-individually similar and different histological subtypes of the analyzed specimen in 49 (87%) and 7 (13%) lesions, respectively.

### Risk factors for recurrence/progression

**Fig. 3:**
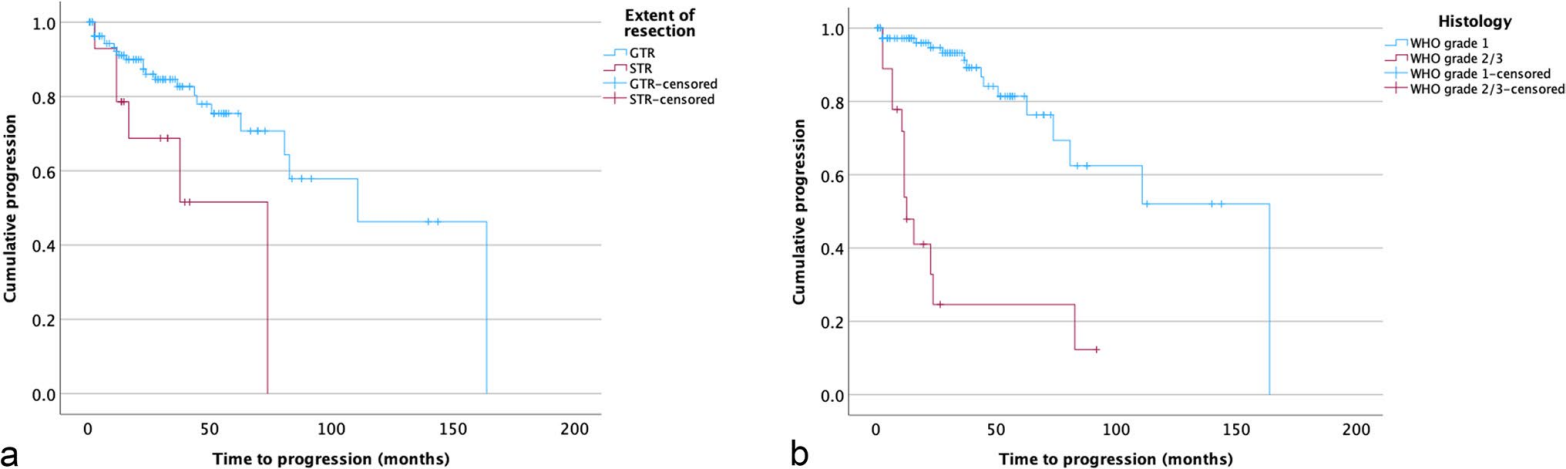
Kaplan–Meier plots illustrating time to progression (TTP) in multiple meningiomas. Median TTP was shorter after STR than after GTR (74 vs 111 months, *p* = 0.014, a) and reduced in grade 2/3 as compared to grade 1 lesions (13 vs 164 months, *p* < 0.001, log rank tests, each)

Within a median follow-up of 56 months (range: 47–64 months), tumor progression occurred in 34 of all operated and non-operated 226 MMs (15%). Progression was observed in 30 of 132 surgically treated lesions but in four of 90 lesions initially subjected to follow-up imaging alone (23% vs 4%, *p* < 0.001). Mean KPS at the date of last follow-up was 70 (SD ± 20) in patients with and 90 (SD ± 20) without tumor progression (*p* = 0.002). Progression was observed in 21 of 180 tumors in females but in 13 of 46 lesions in male patients (12% vs 28%, *p* = 0.010). No correlations were found between progression and age at diagnosis (*p* = 0.662). Progression rates (11% vs 21%, *p* = 0.055) and TTP (medians: 164 months vs not reached, *p* = 0.131) were similar comparing synchronously and metachronously diagnosed meningiomas. Among the 132 operated cases, the risk of progression did not significantly differ comparing GTR and STR (21% vs 43%, *p* = 0.091), while the median TTP was shorter after STR than after GTR (74 vs 111 months, *p* = 0.014, Fig. 3a). Progression was observed in 24 of 113 convexity (21%), 2 of 20 parasagittal/falcine (10%), 7 of 87 skull base (8%), and 1 of 6 spinal meningiomas (17%, *p* = 0.048). Moreover, 72% of the high-grade but 15% of the grade 1 meningiomas developed disease progression (*N* = 13 of 18 vs 17 of 114, *p* < 0.001), and TTP was shorter in grade 2/3 than in grade 1 lesions (13 vs 164 months, *p* < 0.001, log rank test, Fig. 3b). Multivariate, patient-related analyses of the 95 individuals with MM confirmed high-grade histology as the only independent predictor for disease progression (HR 8.11, 95% CI 3.03–21.67; *p* < 0.001, Table 2).

**Table 2:** Multivariate analyses of risk factors for progression in 135 patients with SM (left) and 95 cases with MM (right). Only high-grade histology remained significantly correlated with progression in both cohorts

	Singular meningiomas		Multiple meningiomas	
Variable	HR, 95%CI	*p* value	HR, 95%CI	*p* value
Sex: male vs female (ref.)	1.03, .38–2.82	.509	.93, .25–3.50	.992
Age at surgery (in years)	.90, .96–1.02	.539	1.00, .99–1.05.918	.551
Tumor location: convexity (ref.)	n.s			
Parasagittal	1.91, .59–6.21	.279	1.10, .013–9.54	.933
Skull base	1.38, .44–4.31	.580	.74, .18–3.12	.686
Spinal	1.03, .19–5.49	.972	3.03, .165–55.68	.455
Other	n/a	n/a	n/a	n/a
WHO grade II/III vs I (ref)	3.53, 1.53–8.14	.003	11.59, 3.97–33.86	< .001
Extent of resection: STR vs GTR (ref.)	1.85, .53–6.42	.332	3.09, .820–11.62	.096

### Comparative analyses of patients with MM with SM

Table 1, middle column, summarizes baseline clinical and histopathological data of the cohort of patients with SM (*N* = 135). Frequency of high-grade histology was 7% in skull base meningiomas (*N* = 5 of 76) but 18%, 22%, 13%, and 100% in convexity (*N* = 7 of 38), parasagittal/falcine (*N* = 5 of 23), spinal (N = 1 of 8), and intraventricular (*N* = 1 of 1) tumors, respectively (p = 0.024). High-grade histology was also more commonly found in males (*N* = 13 of 39, 33%, males, vs 4 of 96, 4%, females; *p* < 0.001), and not related with KPS prior index surgery (*p* = 0.174) or at the time of last follow-up (*p* = 0.139).

In comparative patient-related analyses (Table 1, right column), patients with MM were slightly older (75 vs 62 years, *p* = 0.011), while sex distribution (*p* = 0.165), the extent of resection (*p* = 0.576), and the mean KPS at the dates of presentation (*p* = 0.161) and last follow-up (*p* = 0.439) did not significantly differ between patients with SM and MM. Tumor locations differed significantly comparing MM and SM (*p* < 0.001, Table 1). Noteworthy, histopathological analyses revealed a similar distribution of grade 1 and 2/3 histology comparing MM and SM (*p* = 0.857). In contrast to SM, MM were lacking angiomatous, microcystic, psammomatous, chordoid, or anaplastic histology (Table 1).

Within a median follow-up of 78 months (range: 37–252 months), progression was observed in 21% of the patients with SM (*N* = 29). Risk factors for progression in SM are summarized in Table 2, middle column, and were similar compared to MM.

**Fig. 4:**
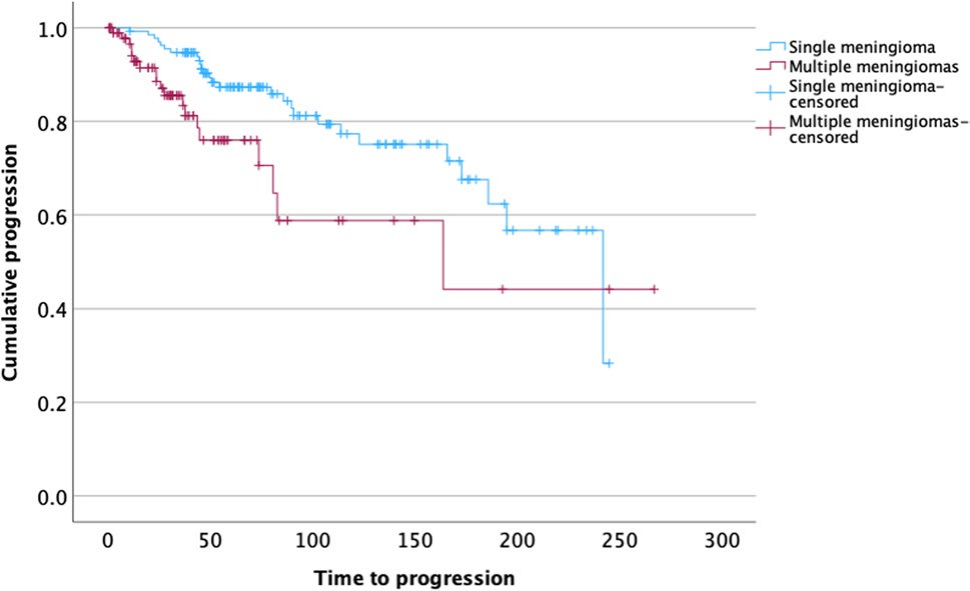
Kaplan–Meier plots comparing time to progression (TTP) in multiple and singular meningiomas. Median TTP was 242 months in singular but 164 months in multiple meningiomas (*p* = 0.011, Log rank test)

Cumulative multivariate analyses *of SM together with MM* adjusted for age, sex, tumor location, the extent of resection, and the WHO grade of the tumors further showed high-grade histology (HR 6.06, 95% CI 3.20–11.47; *p* < 0.001), synchronous or metachronous meningioma growth (HR 4.50, 95% CI 2.26–8.96; *p* < 0.001) and, with borderline significance, STR (HR 2.38, 95% CI 0.97–5.83; *p* = 0.059) as independent predictors for progression. Correspondingly, median TTP was distinctly lower in patients with multiple as compared to cases with singular lesions (164 vs 242 months, *p* = 0.011, Log rank test, Fig. 4). Further Cox regression analyses revealed a similar risk of recurrence in patients with singular and two (HR 1.56, 95% CI 0.76–3.19; *p* = 0.224) meningiomas, but an exponentially increasing risk of progression in patients with 3–4 lesions (HR 3.25, 1.22–1.62; *p* = 0.018) ≥ 5 tumors (HR 13.80, 4.06–46.96; *p* < 0.001).

## Discussion

### Clinical characteristics and histology of patients with MM

Frequency of MM (≥ 2) in our cohort was 7%, which matches the broad range of multiple meningioma incidence reported in previous series [6, 8, 19, 29, 31]. Median age in patients with MM in our series was 62 years and therefore comparable to prior reports [8, 31], but slightly higher as compared to patients with SM. With 79% of the patients, the female predominance in the MM cohort appeared to be higher as in patients harboring SM. Although without reaching the level of statistical significance, this observation matches findings from a previous study [19]. Similar to a former report [31], the majority of MM in our cohort were diagnosed synchronously at the date of initial presentation. As shown in Fig. 1, the frequency of cases with multiple lesions exponentially decreased as the number of tumors increased [31]. MM in our series were found more often in non-skull base locations as compared to SM, contradicting findings of a previous series [19], and further underlying the need of additional studies to define clinical characteristics of MM, regardless of the clinic’s corresponding specialization, e.g., on skull-base surgery. Further clinical characteristics of patients with MM and SM did not significantly differ, consistent with findings of *Maiuri *et al*.* [17].

High-grade histology was found in 13% of all operated MM in our study, which was in line with the control cohort of SM and with rates reported in previous studies on MM [1, 31]. In consideration of the rates of high-grade histology in meningiomas in general, these data suggest a similar distribution of grade 1 and 2/3 tumors in SM and MM. However, by nature, histology was only available in the subgroup of operated tumors, while the WHO grade of lesions treated with irradiation or observation remains obscure. This finding is remarkable as tumor spreading in general is usually assumed to be associated with malignancy and increased proliferation [17]. In contrast to findings in meningiomas in general and to our control cohort of SM [9, 12, 16], high-grade histology in MM was not associated with non-skull base tumor location. Previous studies revealed correlations between molecular characteristics and anatomical distributions of meningiomas in general [16, 22, 28, 34], further raising the questions of genetic and epigenetic differences between SM and MM. Moreover, mean KPS at the date of last follow-up was slightly lower in high-grade than in benign lesions, eventually reflecting disease progression and/or therapy side effects. Although the WHO grade of the tumors remained consistent in most patients, histological subtype differed intra-individually in a considerable portion. In a previous series of seven patients, histological subtype remained identical in specimen from different tumors [6]. In contrast, different histological subtypes of MM in one individual has been reported in other series [8, 31] and contradicts the thesis of direct arachnoid tumor spreading during pathogenesis of MM.

### Prognosis of MM

Progression of MM was observed in 16% and was associated with a reduced KPS at the date of last follow-up. Similar to observations in meningiomas in general, rates of progression were higher in males than in females, eventually reflecting a higher incidence of high-grade histology among the first [24]. Correspondingly, no correlation between patients’ sex and recurrence in patients with MM was found in sex-adjusted multivariate analyses. The impact of the extent of resection on progression in MM is largely unexplored. In our MM cohort, despite similar progression rates, STR was related with shorter progression-free survival, matching findings from *Ramos-Fresnedo *et al. [19]. As this did not hold true in multivariate analyses, the efficacy of GTR on tumor progression in MM remains unclear. Correlation of STR with progression was also lacking in our control cohort of SM, although, in the entire data base, this association has been largely reported [2, 26, 32]. While we cannot exclude a bias, e.g., due to the low number of samples, biological behavior and tumor burden rather than the extent of resection might contribute to tumor progression in MM [3]. Even more than in SM, high-grade histology was found a strong predictor for progression in MM. Considering a lower impact of the extent of resection on progression in these lesions, this finding underlines the necessity of potent adjuvant treatment options in patients with MM and might also display a different biological behavior as compared to SM. This thesis is further underlined by our finding of a more than fourfold increased risk of progression of MM as compared SM. A longer follow-up of patients with SM as compared to patients with MM in our series further supports this theory. Although one might argue that an increasing probability of progression with the number of lesions at risk appears logically, the *exponential* increase is unexpected and contradicts this explanation. Analyses further elucidating underlying molecular alterations in MM, however, remain sparse. Small series and case reports showed mutations or chromosomal losses of NF2 or SMARCB1 in MM, as well as distinct somatic mutations in samples of different meningiomas of one individual [10, 30, 33]. Thus, future analyses are needed to enable molecular characterization of MM and to improve the understanding of their pathogenesis.

The authors are aware of some limitations of the study. Although providing analyses in a large series, our study suffers typical shortcomings from retrospective studies, e.g., selection bias. In addition, complexity of treatment of individual patients with MM causes data heterogeneity and could not be sufficiently considered in statistical analyses. For the same reason, further imaging data could not be subjected to statistical analyses. The number of patients suffering from extensive tumor burden (e.g., > 5 tumors) was low, potentially limiting interpretation of our results. Eventually, treated tumors display different biological behavior and characteristics (e.g., size) as compared to lesions simply subjected to observation, and cumulative analyses might have led to additional bias. Further details about irradiation were not sufficiently available and therefore not subjected to statistical analyses. Finally, samples were neuropathologically diagnosed according to the 2016 WHO classification of brain tumors [18], and molecular characteristics as suggested in the current WHO classification [21] as well as Ki67 labeling index were not considered.

In conclusion, patients with MM were found to lack distinct clinical characteristics as compared to SM. A potential predominant location of MM in non-skull base positions remains to be further investigated. Noteworthy, rates of high-grade tumors did not differ from SM, suggesting alternative underlying, e.g., molecular alterations promoting multiple tumor growth. Risk of progression in MM was generally increased as compared to SM, and exponentially raised with the number of lesions per patient.

## Data Availability

The datasets generated during and/or analyzed during the current study are available from the corresponding author on reasonable request.
